# Antibacterial and Antibiofilm Effects of Allelopathic Compounds Identified in *Medicago sativa* L. Seedling Exudate against *Escherichia coli*

**DOI:** 10.3390/molecules28062645

**Published:** 2023-03-14

**Authors:** Sara Abouzeid, Ulrike Beutling, Engy Elekhnawy, Dirk Selmar

**Affiliations:** 1 Institute for Plant Biology, TU Braunschweig, 38106 Braunschweig, Germany; d.selmar@tu-bs.de; 2 Pharmacognosy Department, Faculty of Pharmacy, Mansoura University, Mansoura 35516, Egypt; 3 Department of Chemical Biology, Helmholtz Centre for Infection Research, 38124 Braunschweig, Germany; ulrike.beutling@helmholtz-hzi.de; 4 Pharmaceutical Microbiology Department, Faculty of Pharmacy, Tanta University, Tanta 31527, Egypt; engy.ali@pharm.tanta.edu.eg

**Keywords:** allelopathic, alfalfa seed exudates, flavonoids, hyperoside, canavanine, dipeptides

## Abstract

In this study, the allelopathic properties of *Medicago sativa* L. (alfalfa) seedling exudates on the germination of seeds of various species were investigated. The compounds responsible for the allelopathic effects of alfalfa were identified and characterized by employing liquid chromatography ion mobility high-resolution mass spectrometry. Crude exudates inhibited the germination of seeds of all various plant species tested. Overall, nine compounds in alfalfa were identified and quantified. The most predominant compounds were a hyperoside representing a flavonoid glucoside, the non-proteinogenic amino acid canavanine, and two dipeptides, identified as H-Glu-Tyr-OH and H-Phe-Glu-OH. The latter corresponds to the first finding that dipeptides are exuded from alfalfa seedlings. In addition, the antibacterial and antibiofilm activities of alfalfa exudate and its identified compounds were elucidated. Both hyperoside and canavanine revealed the best antibacterial activity with minimum inhibitory concentration (MIC) values that ranged from 8 to 32 and 32 to 256 µg/mL, respectively. Regarding the antibiofilm action, hyperoside and canavanine caused a decline in the percentage of *E. coli* isolates that possessed a strong and moderate biofilm-forming potential from 68.42% to 21.05% and 31.58%, respectively. Studies on their inhibiting effects exhibit that these major substances are predominantly responsible for the allelopathic and antimicrobial effects of the crude exudates.

## 1. Introduction

In the course of germination, seeds release various compounds, e.g., coumarins or quinones, which influence the germination, growth, and development of plants growing in the vicinity denoted as allelochemicals [1,2,3]. In addition, these substances might also influence the microbiome, either by the exuded substances inhibiting pathogens and microorganisms that exhibit negative effects for the seedling [4] or by the substances stimulating beneficial bacteria for the seedling [5,6,7,8]

Alfalfa (*Medicago sativa* L.) plants are known to release various allelopathic substances into the soil [9,10,11,12]. Surprisingly, some of these compounds are autotoxic, which means that the plants themselves are impaired by them [13,14,15]. In the case of alfalfa, the allelochemicals are released either from leaves or from the roots [9,13,16,17,18] and include saponins and various isoflavonoids, e.g., medicarpin, 4-methoxymedicarpin, sativan and 5-methoxysativan [14,19,20,21].

Furthermore, seedlings release various flavonoids, such as hyperoside, luteolin, luteolin-7-glucoside, and chrysoeriol [22]. Apart from these phenolic compounds, the exudation of various nitrogen-containing compounds has also been reported for alfalfa seedlings [7,23]. In this context, we consider that these compounds might exhibit a major role in the nodulation process. In this sense, trigonelline and stachydrine from *Medicago* spp. induce *nod* gene expression [7], while the isoflavonoid-derivative medicarpin and the phytoestrogen coumestrol are antagonists of *nod* gene expression [17]. 

In addition, canavanine, which also is exuded from germinating alfalfa seeds, affects the population biology of *Bacillus cereus* UW85 [23]. Furthermore, flavonoid signals from alfalfa seedlings induce the transcription of nodulation (nod) genes in *Rhizobium meliloti* [5,24,25]. However, recently, Compton et al. 2020 [22] reported that the symbiotic bacteria *S. meliloti* is not attracted to any flavonoids identified in alfalfa exudates. Thus, these studies contradict previous investigations. 

As a consequence of the absenteeism of any relevance of the flavanoids for nodulation, their significance in alfalfa seedling exudates has to be scrutinized. It seems reasonable to assume a putative function as allelochemical. Accordingly, this work aims to investigate the allelochemical potential of the compounds exuded from alfalfa seedlings (*Medicago sativa*) and to estimate their impact on the germination of other species. Special emphasis is placed on the verification of an allelopathic function of relevant substances by analysing the impact of *Medicago sativa* seedling exudates on the germination of various seeds. 

Employing liquid chromatography coupled to high-resolution mass spectrometry, the related compounds were identified by comparison with an MS/MS library. Subsequently, seven of these compounds were analyzed concerning their putative allelopathic effects. Finally, the antibacterial and antibiofilm activity of the alfalfa exudate and its identified compounds were evaluated against *Escherichia coli*. 

This bacterium can persist on, enter into and colonize the internal tissues of plants leading to severe food-borne ailments. There are major threats for food safety due to contamination with bacterial pathogens, such as *E. coli* [26]. Apart from the direct influence on human health, there are massive economic losses owing to contaminated food products, which affect many individuals, growers, workers, and distributors [27].

It has to be emphasized that the outcome of this study also entails high relevance for practical applications since allelochemicals have been described to exhibit beneficial implications for sustainable agriculture as alternative herbicides or antimicrobials [28,29].

## 2. Results

### 2.1. Co-Cultivation of Alfalfa Seed with Barley: Impact on Germination and Growth

**Figure 1:**
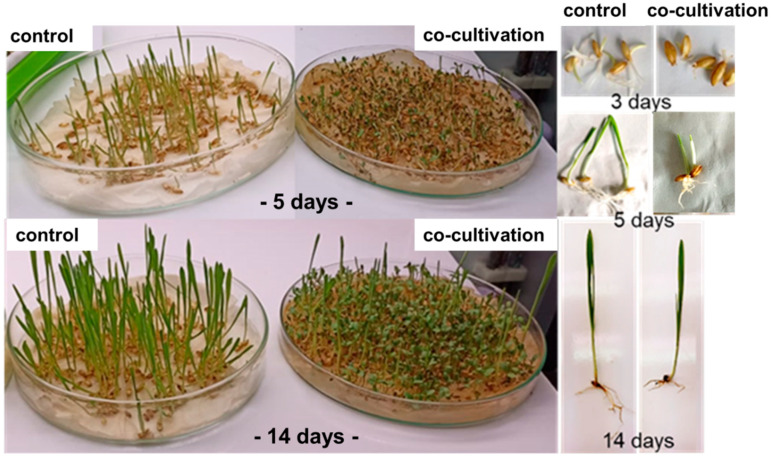
Co-cultivation of alfalfa seed with barley for two weeks. The germination of barley was massively retarded. The delay accounts for about three days in comparison to the controls. In addition, the root and shoot development was significantly reduced in comparison to the controls after 14 days. The experiment was repeated three times.

In order to approve the allelopathic properties of seedling exudates from *Medicago sativa* L., barley (*Hordeum vulgare* L.) seeds were germinated and grown together with alfalfa seeds in the same petri dishes. This co-cultivation strongly impaired the germination and growth of barley (Figure 1):

1. The germination of barley seeds and the subsequent seedling development was massively retarded. The related delay corresponded to up to three days in comparison to the controls.
2. The number of germinated seeds was much lower compared to the controls.
3. The root and shoot development was significantly reduced in comparison to the controls.

### 2.2. Allelopathic Effect of Alfalfa Seed Exudates on Various Plant Species

**Figure 2:**
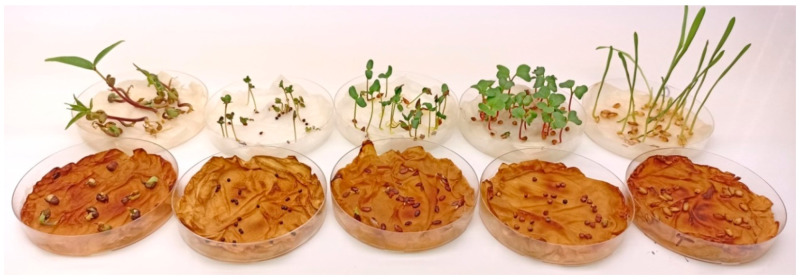
Allelopathic effects of alfalfa seed exudates on various plant species after 10 days. Alfalfa seedling exudate significantly suppressed the seedling growth of all tested plants.

To further analyze the allelopathic potential of *M. sativa* seedlings, a corresponding exudate, i.e., the water, in which the alfalfa seeds had been imbibed for 24 h, was employed. This liquid was used to test the imbibition of seeds of various plant species. Alfalfa seedling exudate significantly suppressed the seedling growth of all tested plants (Figure 2). These data are not suitable to confirm whether the alfalfa seed extracts inhibit germination, if they simply retard it or, alternatively, if the extract is toxic and the seeds died. 

Yet, the latter option of seed death can be excluded, since a transfer of seeds that had been incubated for one week with the root exudates into fresh water resulted in a normal rate of germination. Apart from that, the validation of the allelopathic action does not require differentiation between an inherent inhibition or massive retardation of the germination. Accordingly, no further studies on this topic employing the basic seed extract were performed. The focus of this study was to identify the substances that are responsible for the observed allelopathic effects.

### 2.3. Identification of the Substances Responsible for the Allelopathic Effect in Alfalfa Seeds Exudates

To identify the phytochemical constituents of alfalfa seed exudates, which are responsible for the observed allelopathic effects, the related extract was fractionated by reversed-phase HPLC equipped with a diode array detector. Astonishingly, in the related chromatograms, only three main compounds could be detected. In order to enhance the sensitivity and to perceive substances with no or very low UV absorbance, the detection was extended by utilizing a mass spectrometric detector. Moreover, this approach also generates essential data for the identification of the corresponding substances. 

**Figure 3:**
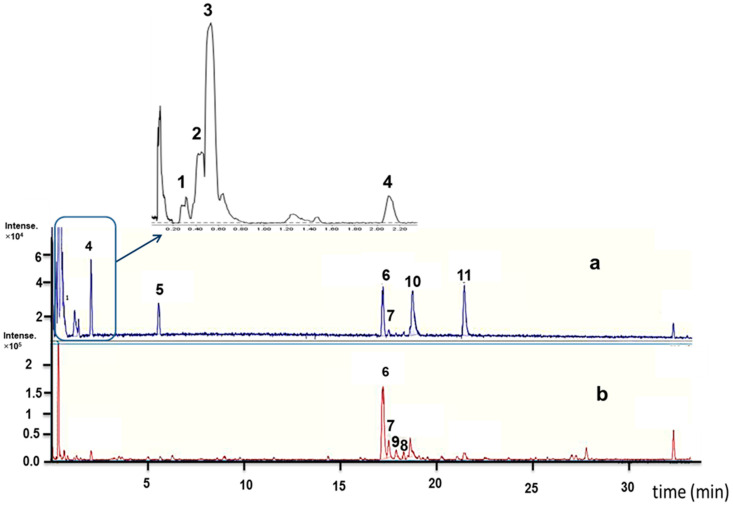
LC-MS base peak chromatogram (BPC) in: (**a**) positive ion mode. (**b**) Negative ion mode of the seedling alfalfa exudate. The peak numbers correspond to the identified compounds listed in Table 1.

**Figure 4:**
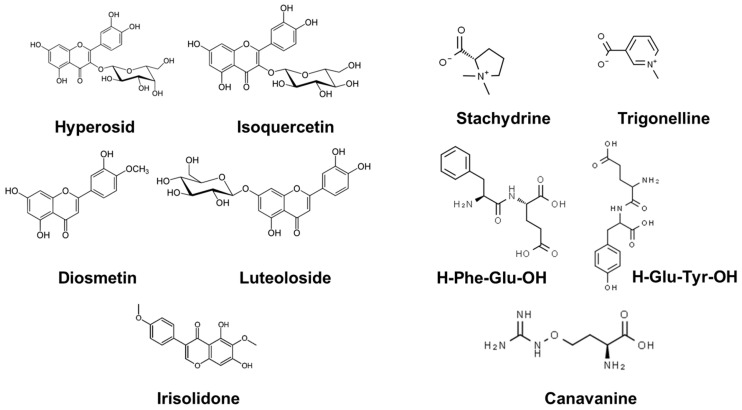
Structure of the main compounds identified through LC–MS/MS in negative mode and positive ESI ion mode.

Using electrospray ionization and high-resolution mass spectrometry in positive and negative ion mode, overall, eleven predominant compounds were detected (Figure 3a,b). By comparing the retention times with authentic standards and their mass spectrometric data with a comprehensive MS/MS library, these substances were unequivocally identified (Table 1 and Figure 4), i.e., as three flavonoid glucosides (hyperoside (Figures S1 and S13, isoquercetin (Figures S2 and S14) and luteoloside (Figures S4 and S15)) as well as five nitrogenous compounds (canavanine (Figure S8), stachydrine (Figure S9), trigonelline (Figure S10) and two dipeptides (H-Glu-Tyr-OH and H-Phe-Glu-OH (Figures S11 and S12)); further details of the structure elucidation are mentioned in the Supplementary Materials). 

Apart from these eight predominant compounds, several minor components were detected e.g., diosmetin (Figures S6, S16 and S17) and an irisolidone isomer (Figures S7 and S18); the related data are also listed in the Supplementary Materials.

### 2.4. Allelopathic Effect of Pure Main Components of the Alfalfa Seed Exudates

To estimate the allelopathic potential of the various constituents of alfalfa, the seven major compounds were purchased and tested for their impact on the germination and seedling development of barley. In the case of H-Glu-Tyr-OH (4), H-Phe-Glu-OH (5), quercetin-3-*O*-xyloside (8), and irisolidone (11), they were not available commercially in the required amounts, and thus no related tests were performed with these compounds.

**Figure 5:**
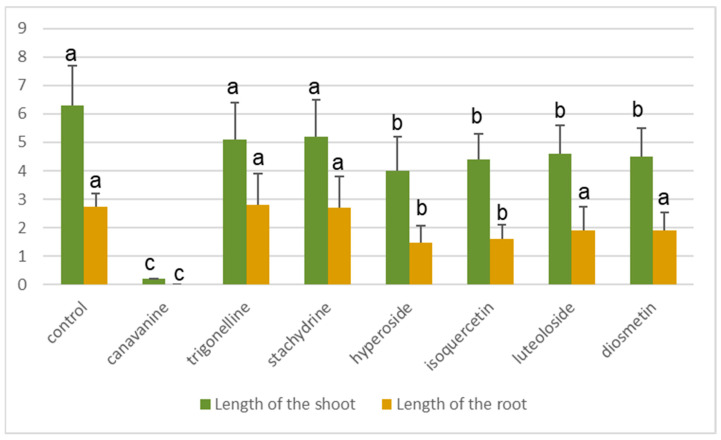
Lengths of the shoots and roots of barley seedlings after treatment with the pure main components of the alfalfa seed exudates after 10 days at a concentration of 150 μg/mL (except for luteoloside, which was employed at 20 μg/mL). Different lowercase letters on the top of the column for each treatment indicate significant differences with *p* < 0.05 using Tukey’s test. The mean value corresponds to the average value of forty seedlings. The error bar corresponds to the standard deviation.

When the seven individual compounds were applied in the same concentration (150 μg/mL; except for luteoloside, which was employed at 20 μg/mL), five of them caused a significant reduction in seedling growth (Figure 5), only stachydrine and trigonelline did not hamper seedling development. The highest and most pronounced allelopathic effect was recorded when canavanine was applied in more or less the same concentration as present in the exudates. 

**Figure 6:**
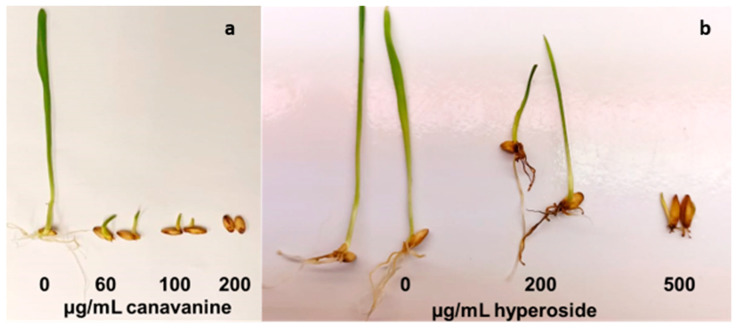
(**a**) Allelopathic effect of canavanine at different concentrations (60, 100, and 200 μg/mL). (**b**) Allelopathic effect of hyperoside at different concentrations (200 and 500 μg/mL) after 10 days.

However, we have to consider that the concentration of the other allelopathic compounds in the exudates may strongly vary, i.e., the concentration of hyperoside in the exudate was more than 1000 µg/mL, whereas that of all the other substances was ten to fifty time lower (Table 2). When the barley seeds were imbibed with water containing hyperoside in half the concentration (500 µg/mL) present in the exudate, the seeds did not germinate at all (Figure 6), and even a concentration of 20% (200 µg/mL) present in the exudate caused massive retardation in seedling development (Figure 6). Accordingly, it can be deduced that the massive inhibition of seedling development by alfalfa seed exudates is mainly due to the action of hyperoside and canavanine.

In addition to the overall retardation of seedling development, a further aspect has to be mentioned, i.e., root growth. Whereas most of the substances may reduce the overall seedling development, canavanine also reduces root development (Figure 5 and Figure 6). Thus, when evaluating the allelopathic potential of a particular substance, especially with respect to the long-term effects, this feature has to be appropriately considered.

One of the most unexpected results concerns a potential autotoxicity of the seedling extracts: when alfalfa seeds were incubated in media containing seedling alfalfa seed exudates, the germination, and growth of the seedling were also significantly retarded in comparison to the control. However, when the seeds were subsequently transferred into the water, germination started, and the seedlings developed normally. Thus, as already mentioned for the seedlings of other species, the exudate is not toxic in *stricto sensu* but inhibits or suppresses regular seedling development. 

Yet, in this context, it has to be noted that, after the transfer of seeds in water, many of the developing seedlings became infected with microbes, whereas under normal conditions, i.e., when the seeds germinated, and in the presence of the exudates, no microbial growth was determined. This suggests that the biological relevance of the related substance—perhaps in addition to their allelopathic effect—might also be due to the prevention of microbial attacks. In this context, the total seedling exudate and its related compounds were tested against bacterial pathogens, such as *E. coli*.

### 2.5. Antibacterial and Antibiofilm Effects of the Alfalfa Seedling Exudate and Its Identified Compounds against Escherichia coli

#### 2.5.1. Sensitivity of *E. coli* Isolates to the Tested Compounds

The total seedling exudate and its compounds exposed antibacterial activity toward *E. coli* isolates using the agar well-diffusion method as there were inhibition zones around the formed wells. Table 3 shows the values of the minimum inhibitory concentrations (MICs) of the total extract and its isolated compounds. Interestingly, hyperoside revealed the best antibacterial activity with MIC values ranging from 8 to 32 µg/mL. This was followed by canavanine (32 to 256 µg/mL) and trigonelline (128 to 256 µg/mL). After that, both luteoloside and the total extract revealed MIC values of 256 to 512 µg/mL. Stachydrine hydrochloride and diosmetin had the highest MIC values of 1024 to 2048 µg/mL.

#### 2.5.2. Antibiofilm Action

Hyperoside and canavanine exposed the highest antibiofilm action toward the biofilm-forming *E. coli* isolates. They caused a decline in the percentage of *E. coli* isolates that possessed a strong and moderate biofilm-forming potential from 68.42% to 21.05% and 31.58%, respectively (Table 4).

#### 2.5.3. Count of Colony-Forming Units per Millilitre (CFU/mL)

**Figure 7:**
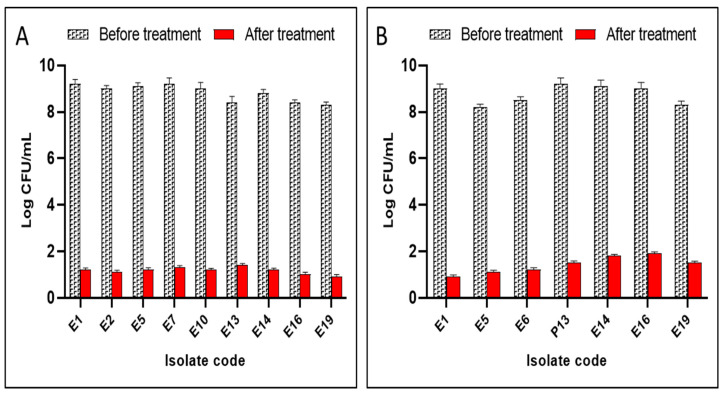
Effects of (**A**) hyperoside and (**B**) canavanine on the count of CFU/mL of the biofilm-forming bacterial cells.

As hyperoside and canavanine showed the best antibiofilm action, the count of CFU/mL was recorded before and after treatment (Figure 7).

## 3. Discussion

In this study, the allelopathic properties of *Medicago sativa* seed exudates on the germination of different seeds were investigated. The compounds responsible for the allelopathic effects of *M. sativa* were analyzed employing liquid chromatography coupled to high-resolution mass spectrometry. The crude exudates inhibited the germination of seeds of all various plant species tested. Overall, eleven compounds in alfalfa were identified and quantified. 

The most predominant compounds were a flavonoid glucoside, identified as hyperoside and non-proteinogenic amino acid canavanine. The results verified that both substances exuded from the alfalfa seeds were predominantly responsible for the allelopathic effect determined for the crude exudates. In this context, it has to be considered that allelopathy comprises a wide array of interactions between plants. 

A corresponding inhibition of competitive plants could be realized either directly, e.g., by inhibiting the germination and growth of potential competitors, or by indirect impacts, e.g., by affecting the microbiome, nutrient availability, and the character of the soil [1]. Alternatively, the compounds exuded from the soils may also be relevant to protect the plants against pathogens that might infest the roots of the seedling. In the next topics, these coherences are discussed for the main compounds exhibiting the major allelopathic effects, i.e., the hyperoside and canavanine exuded from alfalfa seeds.

### 3.1. Hyperoside and Other Flavonoids

As outlined, alfalfa seedlings exude high amounts of hyperoside during the germination as well as various minor flavonoids (e.g., isoquercetin, luteoloside, diosmetin, and irisolidone). The most significant allelopathic effect was attributed to hyperoside—also denoted as quercetin-galactoside. It is well-known that quercetin and its derivatives completely inhibit the growth of *Arabidopsis thaliana* at lower concentrations [30]. Moreover, quercetin (10^−5^ M) is known to be significantly autotoxic and to inhibit alfalfa seed germination [31]. 

Fernández-Aparicio et al., (2021) [32] reported that quercetin showed inhibition of the radicle growth of *Phelipanche ramose*, while apigenin was inactive due to the presence of two *ortho*-free-hydroxy groups of the C ring, such as catechol. High concentrations of quercetin and rutin have been shown to inhibit the seed germination of various plant species by impairing respiration and ATP levels in embryogenic cells via substrate oxidation or phosphate-uptake inhibition, which may uncouple oxidative phosphorylation [31,33]. Additionally, treatment with rutin was shown to drastically reduce the protein content of *Arabidopsis* cells [34].

Apart from these allelopathic effects, flavonoids are also involved in the signaling of alfalfa seedlings to induce the transcription of nodulation (*nod*) genes in *Rhizobium meliloti* [5,25,35]. However, recently, Compton et al., 2020 [22] reported that the symbiotic bacteria *S. meliloti* is not attracted to any flavonoids identified in alfalfa exudates. Thus, these studies contradict previous investigations, and consequently—if the flavanoids do not exhibit any relevance concerning the nodulation—their significance for alfalfa seedlings has to be due to their allelochemical effect by inhibiting the growth of the neighboring plants. 

In addition, other works showed the inhibition role of *P. aeruginosa* biofilm by hyperoside [36]. In the current study, hyperoside had MIC values that ranged from 8 to 32 µg/mL and the highest antibiofilm activity toward the biofilm-forming *E. coli* isolates. Its impact on the viability of the bacterial cells was elucidated via counting the number of CFU/mL of the cells embedded in the biofilm before and after treatment, and the compound revealed a decrease in the count of CFU/mL. Accordingly, the significance of hyperoside in the seed exudates may also be attributed to the protective role against pathogen attack.

### 3.2. Canavanine

L-Canavanine has already been reported as one of the main allelochemicals of jack beans, which strongly inhibits plant growth [37]. The authors also reported that the main mechanism of the allelopathic activity of L-canavanine is related to the suppression of the arginine metabolism in the plant. However, Krasuska et al., (2016) [38] showed that the toxicity of canavanine in tomato roots (*Solanum lycopersicum* L.) is due to alterations in the RNS, ROS, and auxin levels. This effect was not reversible when the seedling was supplemented with arginine (no recovery effect). In addition, L-canavanine at 10 μM suppressed the division of lettuce protoplasts, which was more severe than that of cyanamide [39]. Apart from that, L-canavanine is also described as a natural insecticide. Due to its analogous structure to L-arginine, in most animals, it is falsely loaded to L-arginine-t-RNA since the arginyl-t-RNA synthase does not differentiate between canavanine and arginine. As a consequence, canavanine is incorporated into proteins, resulting in protein malfunctions [40,41].

As already mentioned, plant seeds often exude compounds that impact the population and fitness of the soil microorganism living in the vicinity of the seedlings [42,43]. Our results showed that canavanine sulfate had antibiofilm action toward the biofilm-forming *E. coli* isolates and revealed a decrease in the count of CFU/mL. In this context, it is worth mentioning that canavanine, which is also exuded from germinating alfalfa seeds, is known to affect the population biology of *Bacillus cereus* UW85 [23,44] and, thereby, to increase the chance of the survival of the seed. 

Such coherences were also postulated by Mardani-Korrani et al. (2021) [45] who reported that L-canavanine is exuded from the root of hairy vetch. The authors postulated that canavanine is responsible for altering the soil microbial community and, thus, increases the chance of survival of the vetch seedlings. However, a detailed study by Mardani-Korrani et al. (2021) [45] unveiled that canavanine may either inhibit or stimulate the growth of microorganisms, whereas the bacteria *Firmicutes* and *Actinobacteria* became more abundant in soils after the application of canavanine, *Proteobacteria, and Acidobacteria* populations decreased. 

In addition, Barron and Weaks, (1977) [46] reported a canavanine-mediated enhancement of growth in several isolates of *Pythium* spp. The underlying mechanism of action appears to be the same as reported for the toxic effect of canavanine in insects, i.e., it is falsely incorporated into the microbial proteins [47]. On the other sits, canavanine might be tolerated by other microorganisms and used as a nitrogen source to synthesize other amino acids in the same manner as in the case of the seed beetle *Caryedes brasiliensis,* which reveals the most highly discriminatory arginine-tRNA ligase [48].

Up to now, it is unclear whether the presence of canavanine enhances microbial growth or if the absence of a potential microbicide is the reason for increased microbial attacks. L-canavanine likely influences the interaction of plant seedlings with surrounding organisms in the soil, including soil microbial communities by limiting or stimulating the growth of selected bacteria. However, we still do not know if soil bacteria may use canavanine as a source of nitrogen. 

Furthermore, there is also the possibility that the presence of canavanine may increase the population of soil microorganisms that are capable of utilizing canavanine, which might limit the growth of some competing species [49] and, thereby, act as the typical indirect allelopathic compound. Altogether, canavanine may only be consumed by specific taxa, and it may act as an inhibitor for others. Furthermore, canavanine is also known to be toxic to other plants [37] and, thus, exhibits a direct allelopathic effect. Finally, it might also act as a nitrogen source for other plants.

In conclusion, apart from the allelopathic effect, canavanine clearly also exhibits antimicrobial as well as insecticidal effects. As a consequence, the biological significance of this compound cannot be evaluated by only estimating its allelopathic potential but must also consider its other ecological functions—a typical problem in defining the significance of a natural compound that exhibits various functions. This issue becomes even more complex when considering that canavanine has positive as well as negative effects on the growth of microorganisms. Such ambivalent effects are also known for flavonoids exuded from seeds or roots (see above).

### 3.3. Uptake of the Allelochemicals

In general, allelochemicals might be exported from vital tissues, and depending on the physico-chemical properties, they are either released by passive diffusion or an active exudation by the means of transporters. In this context, allelochemicals, such as coumarins, alkaloids, and phenols, are leached out by simple diffusion across the biomembranes [48]. In contrast, amino acids, such as canavanine, dipeptides, trigonelline, stachydrine and flavonoid glycoside compounds, are unable to simply diffuse across the lipophilic zone of bio-membranes due to their high water solubility. Accordingly, their exudation from alfalfa seedlings requires a carrier, i.e., for their transfer through and across any membrane, transporters are required [50,51,52].

Since any inhibitory effect of an allelochemical requires its uptake, the same coherences also account for their uptake (i.e., the import of polar compounds, such as amino acids and glucosides), which necessitates carriers that can catalyse the membrane transfer. In this context, studies with Arabidopsis (*Arabidopsis thaliana*) mutants atlht1 and ataap1 provided molecular evidence that amino acid transporters facilitate the uptake of amino acids into roots [53,54,55]. 

In addition to amino acids, more complex organic N sources may also be imported by the same mechanism [56]. In addition, Komarova et al., (2008) [57] reported novel evidence that the use of organic forms of N is not restricted to amino acids, and di- and tripeptides should be considered as a potentially important N sources as well. The author showed that AtPTR1 and AtPTR5 transporters facilitate the transport of dipeptides with high affinity, which are localized at the plasma membrane.

Whereas most of the flavonoid aglycones, such as quercetin, reveal a logP of around 2, which expounds their inherent ability to diffuse passively across biomembranes, their glucosidic derivatives are not capable of such diffusion [58,59,60]. 

Even when the logP on some flavonoid glycoside is in the range between −1 and 3, according to the “rule of five”, they cannot cross membranes [59]. Thus, a corresponding import of flavonoidal allelochemicals also requires an appropriate carrier. In contrast, flavonol glycosides can only be transported via ABCC transporters as observed in related in vitro experiments [61,62]. In addition, ABCC transporters are also responsible for transporting flavones and isoflavones into the vacuole [61,63].

Recent studies on the root uptake of organic compounds have shown that allelochemicals exuded into the rhizosphere by plant roots could be absorbed by neighboring plants and translocated into the shoots [2,50,64]. In the case of poisonous specialized metabolites, such as pyrrolizidine alkaloids, this could create massive problems for related plant-derived commodities [50,65,66]. However, it could be argued that the uptake of substances may also imply positive effects, such as acting as protective agents. However, up to now, although this aspect might be particularly interesting for intercropping systems—no sound data on this issue were available.

### 3.4. Beneficial Implications of Allelochemicals for Agriculture

Novel bio-inspired strategies are aimed to replace synthetic herbicides with natural compounds that also inhibit efficiently weed growth [42,67,68,69,70,71]. In this context, allelopathic compounds seem to be the best candidates. As various allelochemicals that inhibit the growth of neighbouring plants are known to be exuded from many legumes, it is suggested to employ them as subsidiary crops: they could either be used as cover crops or in intercropping systems [70,72,73]. However, any living or dead mulch for weed management effective enough to suppress weeds will also hamper the growth of the crop plants and their yield. 

Therefore, research on the employment of allelochemicals has to be focused on balancing the inhibitory effect on weeds and the undesired negative impacts on crop plants; this also accounts for the related effects in the subsequent years [73]. Allelopathic approaches to regulate weed growth include crop rotation, intercropping, cover cropping as living or dead mulches, green manuring, and the use of allelochemical-based bioherbicides [70]. 

In this context, alfalfa can be included in the previous approaches as a prospective and alternative material for friendly environmental weed management to reduce the dependence on synthetic herbicides. Xuan et al., (2005) [68] showed that alfalfa leaf pellets significantly inhibited the germination and growth of four weed species in rice paddies. Furthermore, the paddy field experiment suggested that the dose of 1–2 tons ha^−1^ of alfalfa pellets could control more weed species without any rice plant injury [67,68]. 

In contrast, aqueous extract and crude powder of *Medicago sativa* demonstrated inhibitory effects on the seed germination, growth, and nutrient uptake of *Lycopersicon esculentum* [74]. Weisberger et al., (2019) [75] conducted a meta-analysis across studies involving simple and more diverse crop rotations. They found that diversifying crop rotations, often involving allelopathic crops, such as wheat, oat, corn, alfalfa, and sunflower, reduced weed density by 49% compared to simple sequences, while no significant effects were observed on weed biomass.

### 3.5. Antimicrobial Activity of Allelochemicals against Pathogenic Bacteria

It is essential to investigate novel antimicrobials against multidrug-resistant bacterial isolates. Here, hyperoside had MIC values that ranged from 8 to 32 µg/mL, and this was followed by canavanine (32 to 256 µg/mL). Previous research revealed that hyperoside has antibacterial activity in addition to various other biological potentials, such as anticancer and antioxidant activities [76]. A biofilm is an important virulence factor of *E. coli* pathogenic bacteria. Thus, antibiofilm agents have gained the attention of researchers as a therapeutic trend against biofilm-forming bacteria [76]. In the current study, hyperoside and canavanine had the highest antibiofilm action toward the biofilm-forming *E. coli* isolates. 

Previous research reported that canavanine affects quorum sensing, which is important for biofilm formation [44]. In addition, a previous research article reported the antibiofilm potential of hyperoside [36]. Their impact on the viability of the bacterial cells was elucidated by counting the number of CFU/mL of the cells embedded in the biofilm before and after treatment, and both compounds revealed a decrease in the CFU/mL. This method was chosen as the most accurate, cheapest, and simplest technique that could be used to test the bacterial viability in biofilm after treatment with various compounds [77].

Thus, we can conclude that both hyperoside and canavanine allelochemicals are promising antibacterial and antibiofilm compounds that require further future clinical elucidation.

## 4. Materials and Methods

### 4.1. Chemicals

All solvents used were of HPLC grade and supplied by Fisher Scientific. Canavanine sulfate salt, quercetin-3-glucoside, quercetin dihydrate, hyperoside, luteoloside, stachydrine (hydrochloride), trigonelline chloride, diosmetin, H-Glu-Tyr-OH and H-Phe-Glu-OH standards were purchased from the Sigma-Aldrich (Munich, Germany), Thermo scientific, Carl Roth (Karlsruhe, Germany), Chemodex (St. Gallen, Switzerland), Cayman (Düsseldorf, Germany) and Adipogen life sciences (San Diego, CA, USA) companies.

### 4.2. Co-Cultivation of Barley Seed with Alfalfa Seed

*Medicago sativa* L and barley (*Hordeum vulgare*, L. Poaceae, Sprieβ korngerste, Donath Mühle, Bad Wörishofen, Germany) seeds were surface-sterilized with sodium hypochlorite (0.3%) and rinsed with distilled sterilized water. The seeds were arranged over wetted tissues in petri dishes for germination in the same container under controlled conditions with temperatures ranging from 19 to 22 °C and day/night cycles of 16/8 h. Germination was recorded over 14 days. The experiment was repeated three times.

### 4.3. Preparation of Medicago sativa Seed Exudate

Forty grams seeds of alfalfa were soaked in 500 mL of dist. water for 24 h under aeration. The seed exudate was collected and stored at −20 °C for further experiments.

### 4.4. Evaluation of Allelopathic Effects of Alfalfa Seedling Exudate on Germination

To study the allelopathic effects of alfalfa seedling exudate, different seeds of barley (*Hordeum vulgare* L.), radish (*Raphanus sativus* L.), mung bean (*Vigna radiata* L.), flax (*Linum usitatissimum* L.) and broccoli (*Brassica oleracea* var. italica) were purchased from a local garden center (Reformhaus, Braunschweig, Germany). Thirty seeds of each species were soaked in 50 mL of alfalfa seedling exudate under aeration. After 24 h, the seeds were arranged in petri-dishes lined with two discs of tissues misted with alfalfa seedling exudate for germination under controlled conditions with the temperature ranging from 19 to 22 °C and day/night cycles of 16/8 h, and distilled water was used as a control treatment. Germination was recorded after 10 days at the end of the experiment.

### 4.5. HPLC Analysis of Alfalfa Exudate

Fifty milliliters of alfalfa seedling exudate was freeze-dried, and the residue was dissolved in one milliliter of 80% methanol for HPLC analysis. HPLC was performed using a ReproSil phoenix C18 column (3 μm particle size, L × I.D. 100 mm × 4.6 mm) applying a binary gradient, starting with 85% A, 15% B for 5 min. After 15 min, the ratio was 70% A, 30% B; subsequently, the ratio was changed as follows: 29 min: 50% A, 50% B; 34 min: 20% A, 80% B; 35 min: 20% A, 80% B; 37 min: 85% A, 15% B; and 47 min: 85% A, 15% B using A: aqueous formic acid (1%) and B: acetonitrile formic acid (1%). The flow rate was 0.8 mL/min, and the injection volume was 10 μL. For detection, a photodiode array (PDA) detector was applied at 254, 280, and 350 nm.

### 4.6. Compounds Identification and Quantification Using LC-MS

To identify and quantify the composition of the alfalfa seedling exudate, high-resolution UPLCMS analysis was performed using a Bruker TimsTOFPro mass spectrometer, equipped with an Apollo II Elektrospray source. For UHPLC, a Dionex Ultimate3000RS from Thermo Scientific, Dreieich, Germany was used for the separation, containing an autosampler, binary high gradient pump, column oven, 6-port-column-switching-option, and DAD-detector. 

The column used for the separation: Aquity UPLC CSH Phenyl-Hexyl; 1.7 µm; 2.1 × 50 mm, from Waters, column temperature: 40 °C, UV spectra recording: 190—600 nm, and injection volume: 2 µL. Solvents: A: Water with 0.1% formic acid, B: Acetonitrile with 0.1% formic acid, flow rate: 500 µL/min. Used gradient: 0 min 0% B, 2 min 0% B, 10 min 5% B, 30 min 30% B, 50 min 100% B, and 55 min 100% B. After this wash, the column with 100% B, and then return to the starting conditions with 0% B. The overall run time was 60 min. MS conditions: within the first 0.3 min of the run, a mixture of low-concentration Tune-Mix and sodium formate was infused as calibrant to the system (for internal calibration and mobility calibration). 

Calibrations were performed in Data Analysis (internal and mobility calibration—Calibration Mode HPC for mass, linear for mobility). MS Acquisition Parameter (adapted from the method, delivered with the device control system Otof-control v6.2 from Bruker, Billerica, MA, USA): source type: ESI, scan range: 20–1000 *m*/*z*, ion polarity: positive/(negative), capillary voltage: 4500 V, nebulizer pressure: 2.2 bar, dry heater: 220 °C, dry gas: 10.0 l/min. PASEF/TIMS: TIMS ramp time: 100 ms, spectra rate: 9.52 Hz, number of PASEF MSMS scans: 2, total cycle time: 0.53 s. Mobility: 1/K0 scanned from 0.45 to 1.20 V*S/cm^2^. 

The concentrations employed for the calibration curves ranged from 50 µg/mL to 200 mg/mL. For reliable identification and quantification, authentic reference substances were applied.

### 4.7. Allelopathic Effect of Pure Main Components of the Alfalfa Seed Exudates

Hyperoside, canavanine, trigonelline, stachydrine, isoquercetin, luteoloside and diosmetin were tested for allelopathic effects on barley seeds. Barley seeds were immersed in an aqueous solution at a concentration of 150 μg/mL (except for luteoloside, which was employed at 20 μg/mL). After 24 h, the seeds were arranged in Petri dishes lined with two tissues misted with an aqueous solution of different compounds for germination under controlled conditions with temperatures ranging from 19 to 22 °C and day/night cycles of 16/8 h. The shoot length was recorded after 10 days at the end of the experiment. In addition, barley seeds were immersed in hyperoside aqueous solution at concentrations of 200 and 500 μg/mL and canavanine at 60, 100 and 200 μg/mL. The shoot and root lengths were recorded after 10 days at the end of the experiment.

### 4.8. Antibacterial and Antibiofilm Effects of the Alfalfa Seedling Exudate and Its Identified Compounds against Escherichia coli

#### 4.8.1. Bacteria

Nineteen *E. coli* isolates were used in the current study. They were clinical isolates obtained from the Department of Pharmaceutical Microbiology, Faculty of Pharmacy, Tanta University. These bacterial isolates were multidrug-resistant, and their resistance profile is shown in Table S1.

#### 4.8.2. Antibacterial Action

The susceptibility of *E. coli* isolates to the total extract and its isolated compounds was revealed by the agar well-diffusion method [78]. Briefly, after spreading the bacterial suspensions on the Muller–Hinton agar plates, cups were made, and the tested compounds, in dimethyl sulfoxide (DMSO) with concentrations of 1000 µg/mL, were poured into the cups. A positive control (ciprofloxacin) and a negative control (DMSO) were incorporated.

#### 4.8.3. Determination of MICs

The MIC values were recorded using the broth microdilution method [79] in 96-well plates. The tested compounds were two-fold serially diluted using Muller–Hinton broth in the wells of the microtitration plates, and the bacterial suspensions were then added. The tested plates were incubated overnight at 37 °C, and the MIC values were recorded as the minimum concentration of the compound that inhibited the bacterial growth.

#### 4.8.4. Antibiofilm Action

The influence of the tested compounds was revealed on the capacity of biofilm formation by *E. coli* isolates (at their 0.5 MIC values) using a crystal violet assay [80,81]. The optical density (OD) values were elucidated at 490 nm by a microtitration plate reader. The capacity of the biofilm formation was revealed, and it was grouped into four groups as follows: non-biofilm, weak, moderate, and strong biofilm formation [82].

#### 4.8.5. Colony-Forming Units (CFU/mL) Count

After allowing the isolates to form biofilms in the wells of the microtitration plates, they were thoroughly washed using phosphate buffered saline (PBS), and they were scraped off the wells after adding 200 μL PBS. They were then mixed well and diluted. About 100 μL of the suspension was inoculated into Muller–Hinton agar plates, which were then incubated overnight at 37 °C. After incubation, the CFU/mL count was recorded [83].

### 4.9. Statistical Analyses

Each experiment was performed in triplicate, and the data are expressed as the mean ± standard deviation. Quantitative data were statistically analyzed using one-way analysis of variance (ANOVA). Significant differences between the obtained data were determined using Tukey’s test, with *p* < 0.05 implying statistical significance. Statistical analyses were performed using SPSS v20.0 from SPSS Inc. (Chicago, IL, USA) and GraphPad Prism 8 (GraphPad Software Inc., San Diego, CA, USA). 

## 5. Conclusions

The most active allelopathic compounds exuded from alfalfa seeds were hyperoside and canavanine, which were predominantly responsible for the inhibition of germination by crude exudates. Allelopathic effects could be due to a wide array of effects. Accordingly, apart from the inhibition of germination, their allelopathic action may also be due to affecting the microbiome, the nutrient availability, and the character of the soil. A related study on these putative effects is pending. Furthermore, the compounds exuded into the soil may also be relevant to protect the plants against pathogens, which might infest the roots of the seedling.

Special emphasis has to be placed on the antimicrobial activity of allelochemicals against pathogenic bacteria, e.g., *E. coli*, which opens the intriguing possibility to study the effects of allelopathic compounds, i.e., hyperoside and canavanine, as promising antibacterial and antibiofilm candidates that require further future clinical elucidation.

Overall, the allelopathic properties of the alfalfa-derived flavonoids, as well as canavanine, suggest their high potential as bioherbicides, which could be employed in organic farming.

## Figures and Tables

**Table 1:** Characterization by UPLC-ESI-QTOF-MS/MS of the compounds present in the alfalfa exudates.

#	Name	Rt. (min)	CCS	*m*/*z* Meas, [M + H]^+^	Molecular Formula	MS/MS Fragments
**Nitrogenous compounds**						
1	Canavanine	0.29	134.6	177.09781	C_5_H_12_N_4_O_3_	76, 72, 61, 56, 44
2	Trigonelline	0.41	122.3	138.05495	C_7_H_7_NO_2_	138, 92, 78, 65, 51, 39
3	Stachydrine	0.5	126.6	144.101905	C_7_H_13_NO_2_	144, 102, 84, 72, 58, 44
4	H-Glu-Tyr-OH	2.1	171.9	311.12306	C_14_H_18_N_2_O_6_	311, 248, 202, 182, 165, 147, 136, 123, 103, 91, 84
5	H-Phe-Glu-OH	5.6	169.4	295.1285	C_14_H_18_N_2_O_5_	295, 233, 186, 166, 149, 120, 103, 84
**Flavonols and Flavonol glycosides**						
6	Quercetin 3-galactoside (hyperoside)	17.26	205	465.1022	C_21_H_20_O_12_	465, 303, 257, 229, 153, 85
7	Quercetin 3-*O*-glucoside (isoquercetin)	17.58	206.9	465.10222	C_21_H_20_O_12_	465, 303, 257, 229, 153, 85
8	Quercetin 3-*O*-xyloside	18	182	435.05743	C_20_H_18_O_11_	435, 303, 257, 229,153
**Flavones and flavones glycosides**						
9	Luteoloside	18.0	211.5	449.2856	C_21_H_20_O_11_	449, 287, 258, 153
10	Diosmetin isomer	18.8	167.9	301.07006	C_16_H_12_O_6_	301, 286, 269, 258, 229, 213, 184, 153, 124, 96
**Isoflavone**						
11	Irisolidone isomer	21.5	172.5	315.08569	C_17_H_14_O_6_	315, 300, 272, 257, 229, 167, 148, 133

**Table 2:** The concentration of compounds present in the alfalfa seedling exudates.

Compound	Concentration (µg/mL)
Canavanine	160.9
Trigonelline	54.1
Stachydrine	70.9
Hyperoside	1134
Isoquercetin	29.9
Luteoloside	1.26
Diosmetin	11.4
H-Glu-Tyr-OH	275
H-Phe-Glu-OH	14.9

**Table 3:** Minimum inhibitory concentration values of the total extract and its isolated compounds.

Isolate Code	MIC Values (µg/mL)						
	Total Exudate	Canavanine	Luteoloside	Hyperoside	Stachydrine	Diosmetin	Trigonelline
E1	512	256	256	8	1024	1024	128
E2	512	128	256	8	1024	2048	128
E3	256	256	256	16	1024	1024	128
E4	256	128	256	8	2048	1024	256
E5	512	32	512	32	1024	1024	256
E6	512	64	512	16	1024	1024	128
E7	512	64	512	32	1024	2048	256
E8	512	256	256	16	2048	1024	128
E9	256	256	256	8	2048	1024	128
E10	256	32	256	16	1024	2048	128
E11	256	64	512	32	1024	1024	128
E12	512	64	256	16	1024	1024	256
E13	512	32	512	32	2048	1024	256
E14	256	64	512	8	2048	2048	128
E15	512	128	512	16	1024	1024	128
E16	256	128	512	32	1024	2048	256
E17	256	256	256	32	1024	1024	256
E18	256	128	256	32	1024	1024	128
E19	256	64	256	16	1024	1024	256

**Table 4:** Impact of the tested compounds on the biofilm of *E. coli* isolates.

Biofilm Formation Ability	Count of *E. coli* Isolates before Treatment	Count of *E. coli* Isolates after Treatment with						
		Total Exudate	Canavanine	Luteoloside	Hyperoside	Stachydrine	Diosmetin	Trigonelline
None forming	2	3	4	3	4	2	2	2
Weak	4	6	9	4	11	5	6	5
Moderate	7	6	3	6	3	7	6	6
Strong	6	4	3	6	1	5	5	6

## Data Availability

Not applicable.

## Supplementary Materials

The following supporting information can be downloaded at: https://www.mdpi.com/article/10.3390/molecules28062645/s1, Figures S1–S18: MS/MS spectra. Table S1. Resistance profile of the tested isolates.
