# Supplementary material for: Antibacterial and Antibiofilm Effects of Allelopathic Compounds Identified in Medicago sativa L. Seedling Exudate against Escherichia coli
Source: Molecules. 2023 Mar 14;28(6):2645. doi: 10.3390/molecules28062645 (PMC10056293; doi:10.3390/molecules28062645)
Supplement: Supplementary file 1 [file molecules-28-02645-s001.zip › molecules-2262506-supplementary.pdf]

Antibacterial and Antibiofilm Effects of Allelopathic Compounds Identified in *Medicago sativa* L. Seedling Exudate Against *Escherichia coli*

Sara Abouzeid, Ulrike Beutling, Engy Elekhnawy, Dirk Selmar

Analysis in the Negative-ion Mode of alfalfa exudate:

Identifcation of flavonoids:

Flavonols and Flavonol glycosides

Quercetin glycosides were identified in the extract. **Compound at Rt 17.3** showed a molecular ion peaks $[M-H]^-$ at *m/z* 463.06672, and was identified as quercetin 3-galactoside in comparison with hypersoide standard (Fig. S1, S13). **Compound at Rt 17.6** showed the similar a molecular ion peaks $[M-H]^-$ at m/z 463.06672, MS/MS spectrum and CCS (Ion mobility collision cross-section) of compound at Rt 17.3. This compound was identified as a quercetin 3-O-glucoside (Fig. S2, S14). **Compound at Rt 18.7** showed a molecular ion peaks $[M-H]^-$ at *m/z* 433.0743, in which glycoside were cut out from their structures with pentose sugar moiety (*m/z* 133). This compound was tentatively identified as Quercetin 3-O-arabonoside, arabinofuranoside, or xyloside (Fig. S3).

Flavones and flavones glycosides

Luteoloside was identifed at peak with **Rt 18 mint**, showing a molecular ion peak [M-H]− at *m/z* 447.0923 and base peak at *m/z* 285.0772 corresponding to **luteoloside** and it's aglycone (Fig. S4, S15).

Compound at Rt 18.8 showed a molecular ion peaks $[M-H]^-$ at *m/z* 299.056243, and was identified as luteolin methyl ether i.e diosmetin (Fig. S6, S16, S17)

Compound at Rt 21.5 showed a molecular ion peaks $[M-H]^-$ at *m/z* **313.0565**, and was identified as irisolidone isomer (Fig. S7, S18)

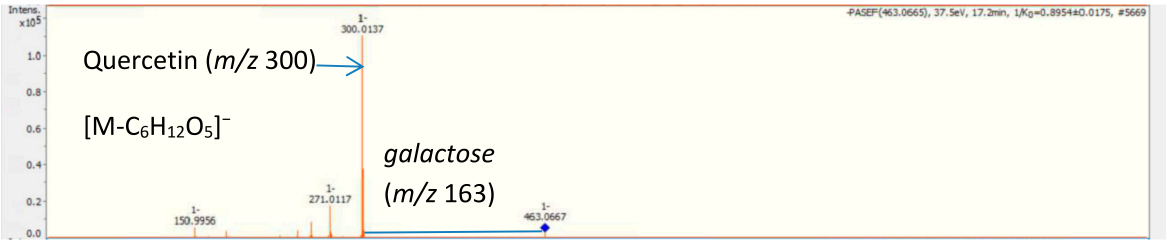

Figure S1: MS/MS spectrum of quercetin 3-galactoside at Rt 17.2with $[M\text{-}H]^-$ at *m/z* 463.06672

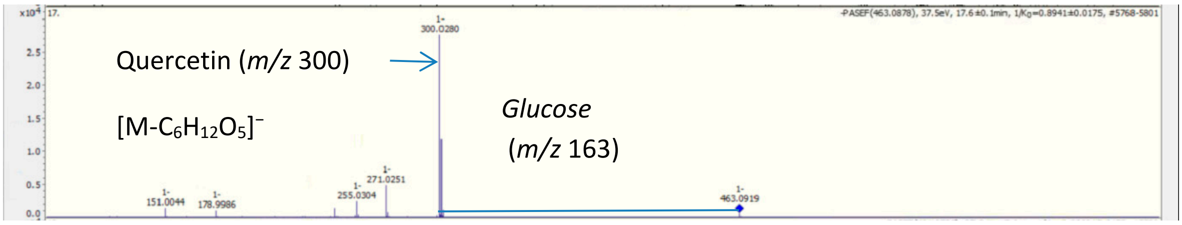

Figure S2: MS/MS spectrum of quercetin 3-O-glucoside at Rt 17.6 with $[M\text{-}H]^-$ at *m/z* 463.0919

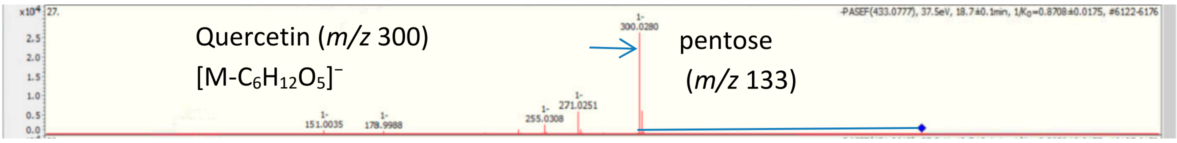

Figure S3: MS/MS spectrum of quercetin 3-O-xyloside at Rt 18.7 with [M-H]– at *m/z* 433.05743

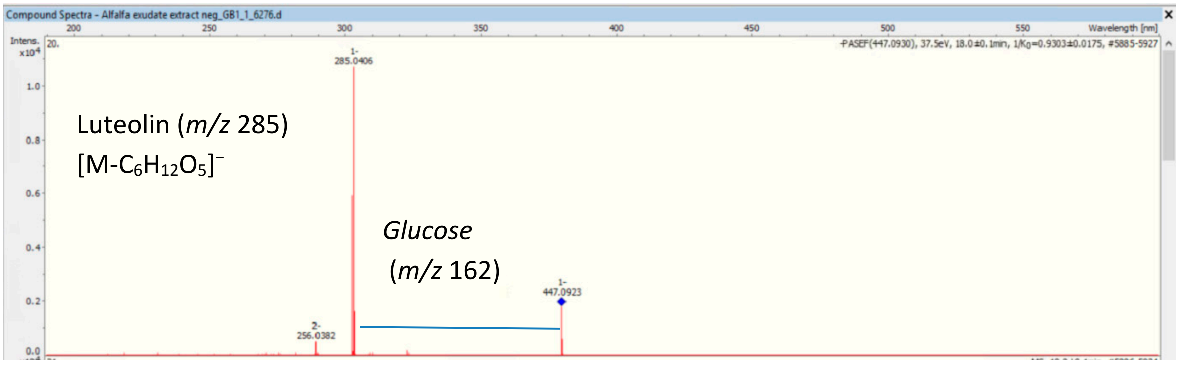

Figure S4: MS/MS spectrum of luteoloside at Rt 18 mint with [M-H]– at *m/z* 447.0923

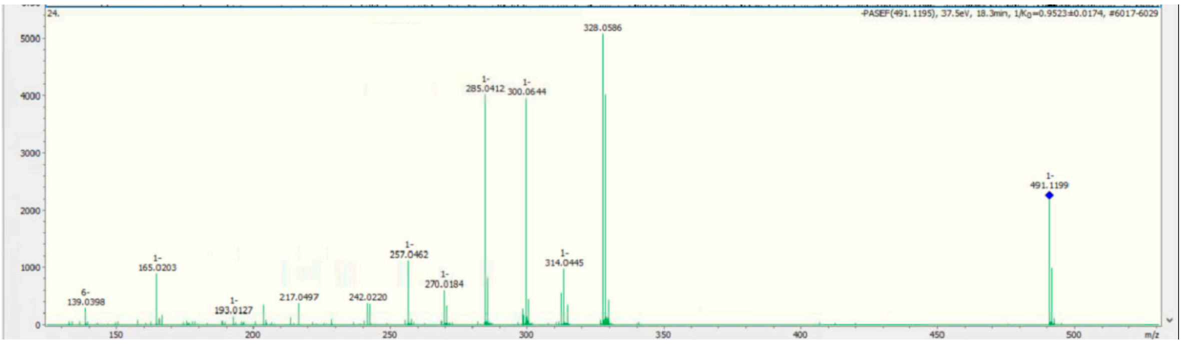

Figure S5: MS/MS spectrum of Compound at Rt 18.3 mint with $[M\text{-}H]^{-}$ at *m/z* 491.09722 (unknown)

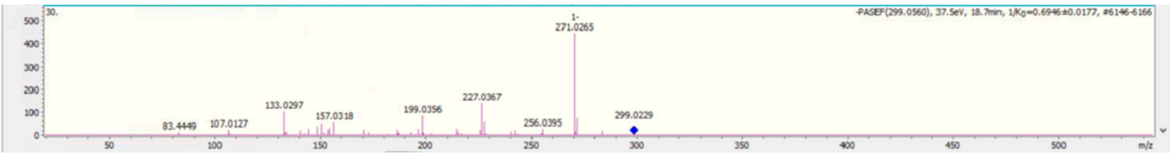

Figure S6: MS/MS spectrum of diosmetin isomer at Rt 18.7 mint with $[M\text{-}H]^{-}$ at *m/z* 299.0229 .

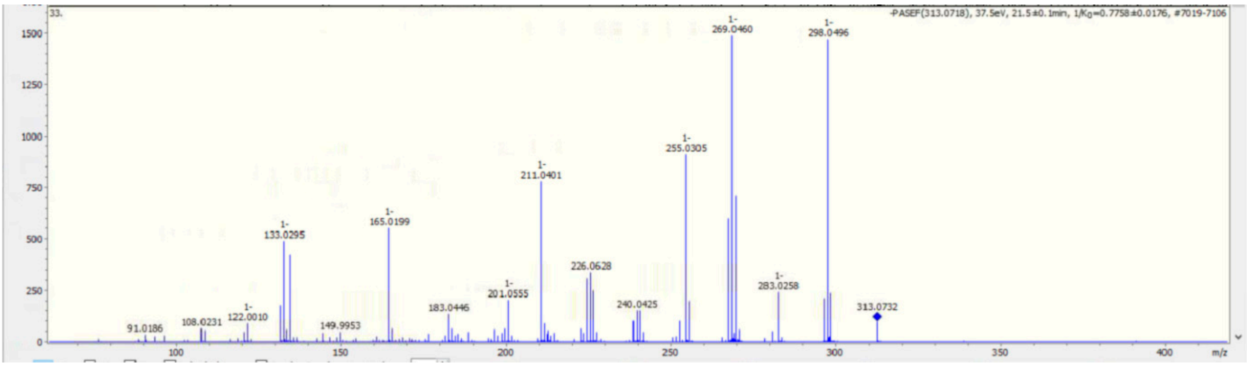

Figure S7: MS/MS spectrum of irisolidone isomer at Rt 21.5 mint with $[M\text{-}H]^{-}$ at *m/z* 313.0732

• Analysis in the Positive-ion Mode

Identification of nitrogenous compounds:

From the analytical perspective, it is clear that amino acids are not detectable by HPLC–UV analysis due to the lack of a strong chromophore and that HPLC analysis of all compounds requires gradient elution. Mass spectrometry represents an effective detection method, and improvement in

selectivity and specificity can be raised by using tandem mass ion mobility mass spectrometry. All compounds were confirmed by comparison with commercial standards.

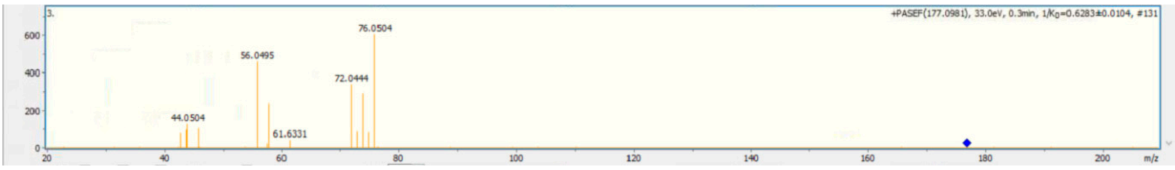

Figure S8: MS/MS spectrum of canavanine at Rt 0.3 mint with $[M+H]^+$ at *m/z* 177.0981

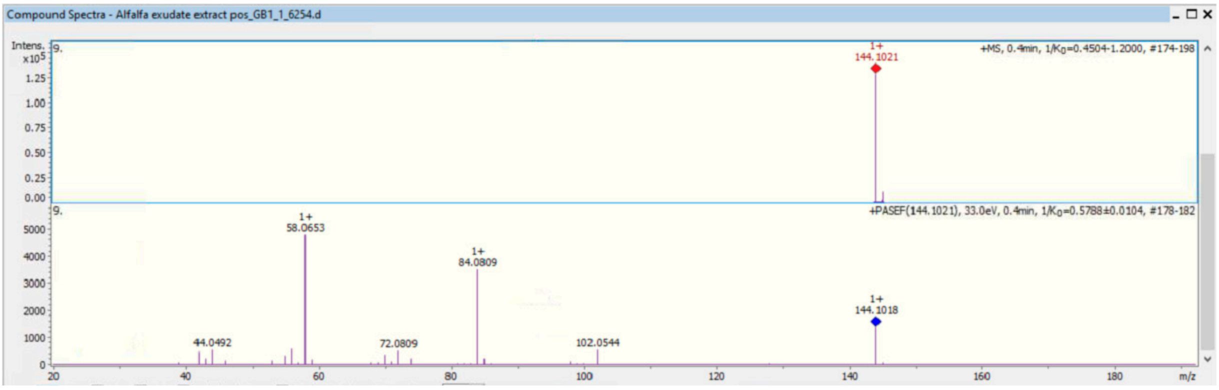

Figure S9: MS/MS spectrum of stachydrine at Rt 0.4 mint with $[M+H]^+$ at *m/z* 144.1018

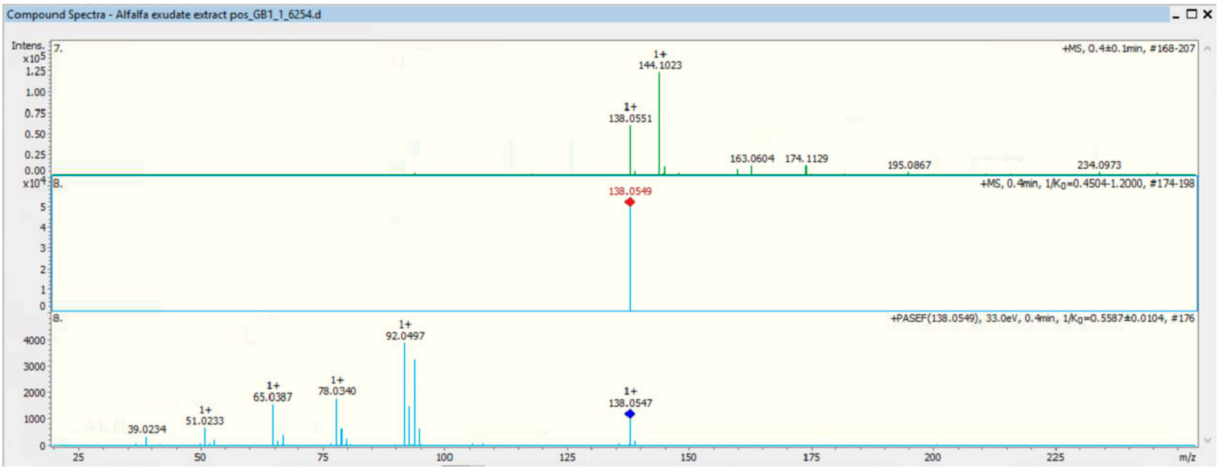

Figure S10: MS/MS spectrum of trigonelline at Rt 0.4 mint with $[M+H]^+$ at *m/z* 138.0547

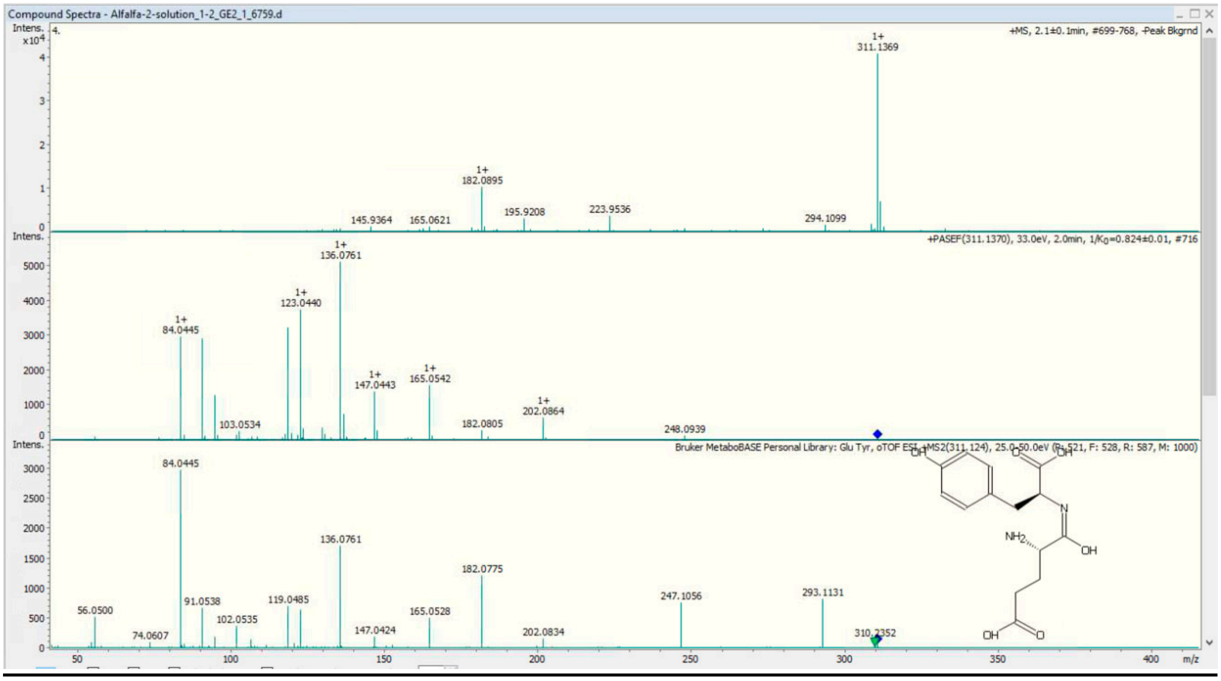

Figure S11: MS/MS spectrum of Glu-Tyr dipeptide at Rt 0.4 mint with $[M+H]^+$ at *m/z* 311.1370

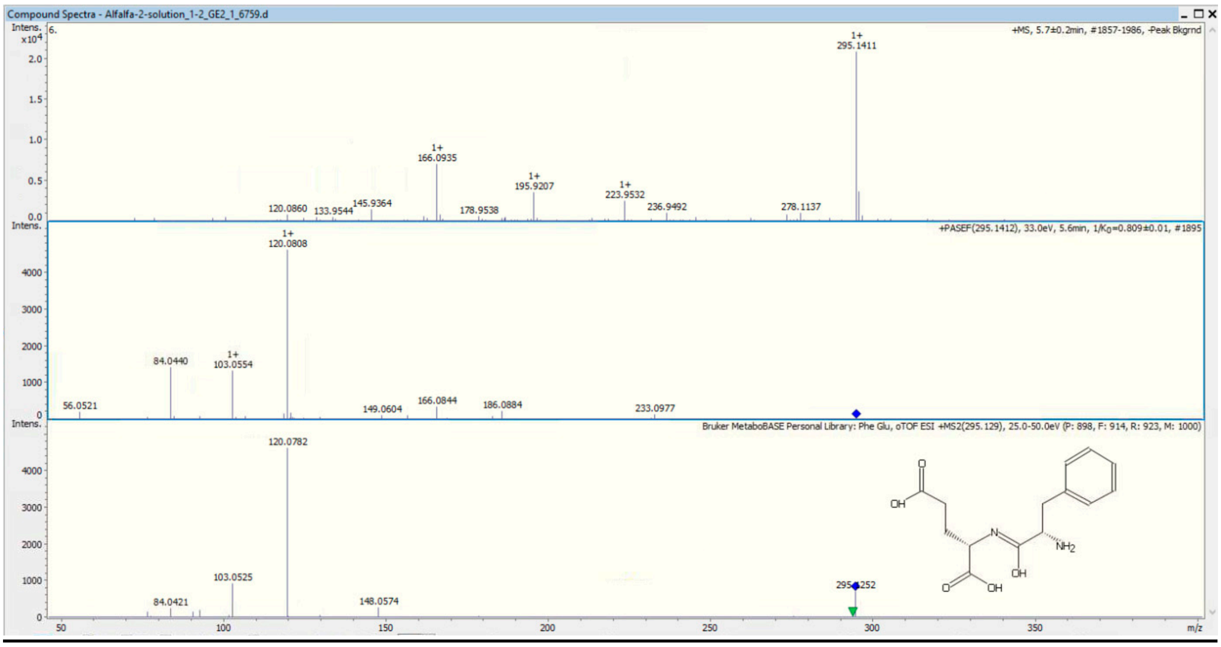

Figure S12: MS/MS spectrum of Phe-Glu dipeptide at Rt 5.6mint with $[M+H]^+$ at *m/z* 295.1412.

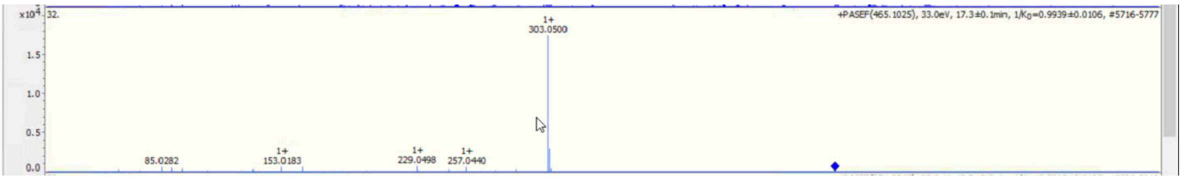

Figure S13: MS/MS spectrum of Compound at Rt 17.3 mint with $[M+H]^+$ at *m/z* 465.1025 (hypersoide)

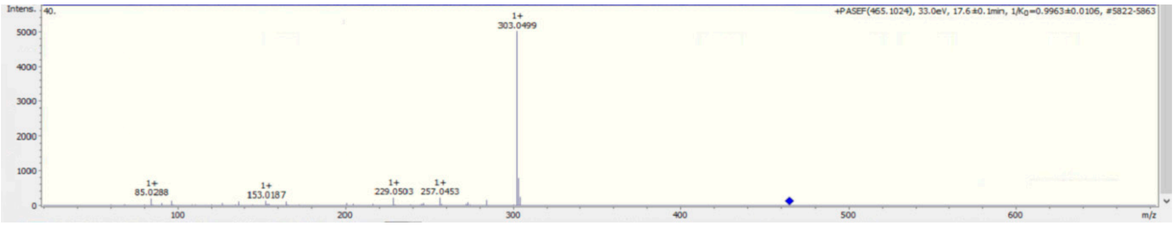

Figure S14: MS/MS spectrum of Compound at Rt 17.6 mint with $[M+H]^+$ at *m/z* 465.1024 **(**isoquercetin)

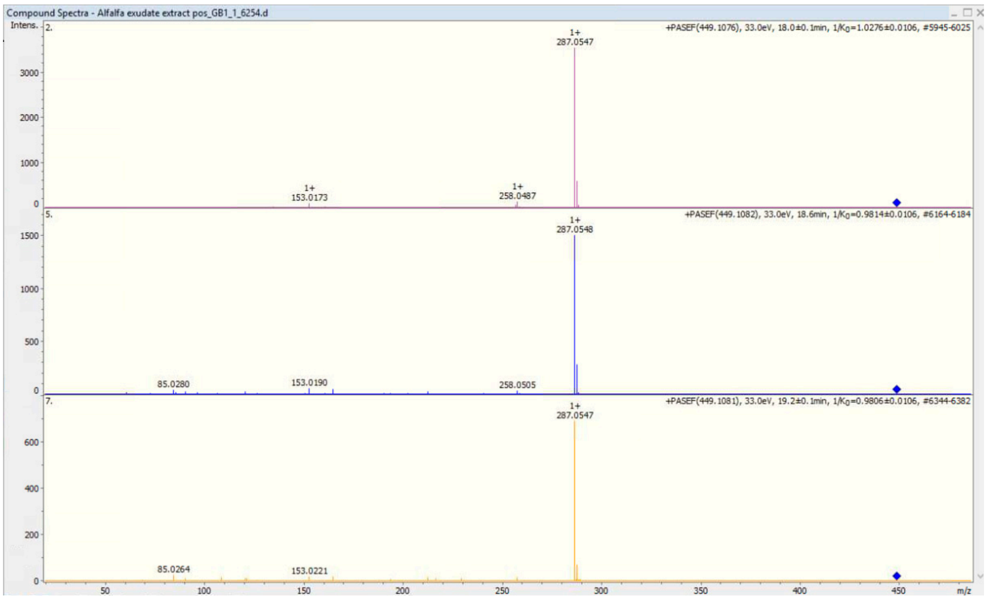

Figure S15: MS/MS spectrum of Compound at Rt 18 mint with $[M+H]^+$ at *m/z* 499.108 in comparison with luteoloside standard

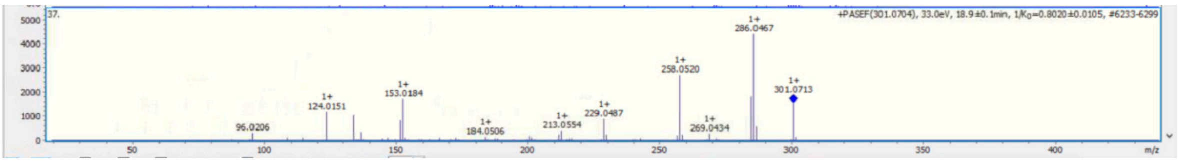

Figure S16: MS/MS spectrum of Compound at Rt 18.9 mint with $[M+H]^+$ at *m/z* 301.0704 (diosmetin isomer)

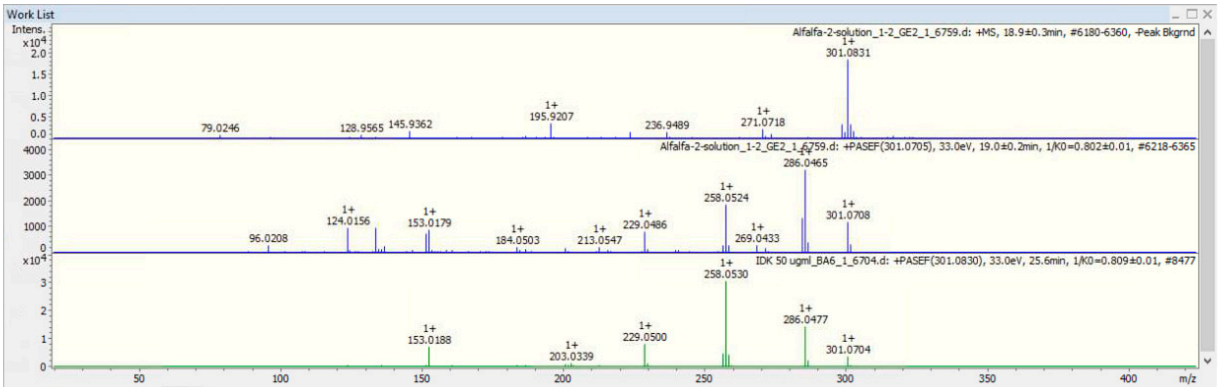

Figure S17: MS/MS spectrum of Compound at Rt 18.9 mint with $[M+H]^+$ at *m/z* 301.0704 in comparison with diosmetin

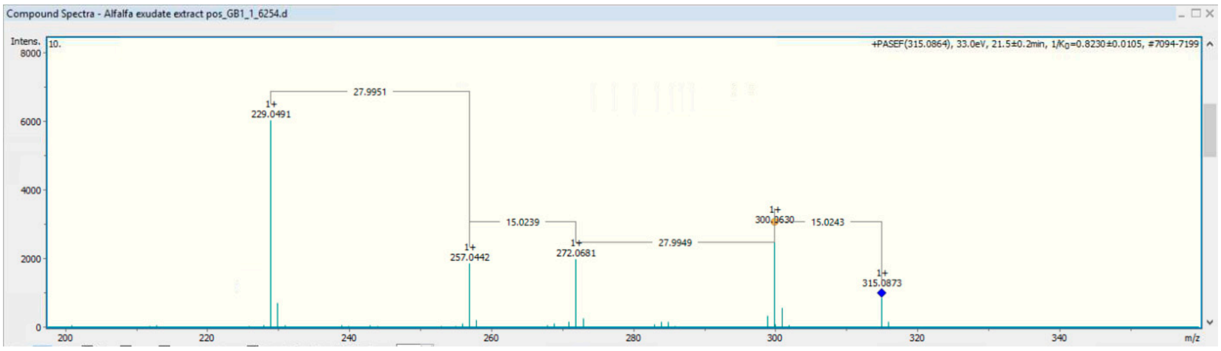

Figure S18: MS/MS spectrum of irisolidone isomer at Rt 21.6 mint with $[M+H]^+$ at *m/z* 315.0875.

Table S1. Resistance profile of the tested isolates.

Isolate code	Resistance profile
E1	Amoxicillin-sulfamethoxazole/trimethoprim- tetracycline
E2	Amoxicillin-tetracycline-amikacin
E3	Amoxicillin-ampicillin/sulbactam-amikacin
E4	Amoxicillin-tetracycline- amikacin
E5	Ampicillin/sulbactam- sulfamethoxazole/trimethoprim- tetracycline-amikacin
E6	Amoxicillin- ampicillin/sulbactam- sulfamethoxazole/trimethoprim-tetracycline-amikacin
E7	Amoxicillin- sulfamethoxazole/trimethoprim- tetracycline-amikacin
E8	Amoxicillin- tetracycline-amikacin
E9	Amoxicillin- ampicillin/sulbactam- sulfamethoxazole/trimethoprim-tetracycline
E10	Amoxicillin-tetracycline-amikacin
E11	Amoxicillin- ampicillin/sulbactam- tetracycline
E12	Amoxicillin- ampicillin/sulbactam- sulfamethoxazole/trimethoprim-tetracycline-ciprofloxacin
E13	Amoxicillin- ampicillin/sulbactam- tetracycline-amikacin
E14	Amoxicillin- sulfamethoxazole/trimethoprim- tetracycline
E15	Amoxicillin- ampicillin/sulbactam- tetracycline-amikacin
E16	Amoxicillin- ampicillin/sulbactam- sulfamethoxazole/trimethoprim-tetracycline
E17	Amoxicillin- ampicillin/sulbactam- amikacin
E18	Amoxicillin-sulfamethoxazole/trimethoprim- tetracycline-amikacin
E19	Amoxicillin- ampicillin/sulbactam- sulfamethoxazole/trimethoprim
